# FB5P-seq: FACS-Based 5-Prime End Single-Cell RNA-seq for Integrative Analysis of Transcriptome and Antigen Receptor Repertoire in B and T Cells

**DOI:** 10.3389/fimmu.2020.00216

**Published:** 2020-03-03

**Authors:** Noudjoud Attaf, Iñaki Cervera-Marzal, Chuang Dong, Laurine Gil, Amédée Renand, Lionel Spinelli, Pierre Milpied

**Affiliations:** ^1^Aix Marseille Université, CNRS, INSERM, Centre d'Immunologie de Marseille-Luminy, Marseille, France; ^2^Centre de Recherche en Transplantation et Immunologie UMR1064, INSERM Université de Nantes, Nantes, France

**Keywords:** single-cell RNA sequencing, transcriptome, antigen receptor, B cells, T cells

## Abstract

Single-cell RNA sequencing (scRNA-seq) allows the identification, characterization, and quantification of cell types in a tissue. When focused on B and T cells of the adaptive immune system, scRNA-seq carries the potential to track the clonal lineage of each analyzed cell through the unique rearranged sequence of its antigen receptor (BCR or TCR, respectively) and link it to the functional state inferred from transcriptome analysis. Here we introduce FB5P-seq, a FACS-based 5′-end scRNA-seq method for cost-effective, integrative analysis of transcriptome and paired BCR or TCR repertoire in phenotypically defined B and T cell subsets. We describe in detail the experimental workflow and provide a robust bioinformatics pipeline for computing gene count matrices and reconstructing repertoire sequences from FB5P-seq data. We further present two applications of FB5P-seq for the analysis of human tonsil B cell subsets and peripheral blood antigen-specific CD4 T cells. We believe that our novel integrative scRNA-seq method will be a valuable option to study rare adaptive immune cell subsets in immunology research.

## Introduction

Technologies to reliably amplify and sequence the mRNA content of single cells have progressed dramatically. By quantitatively measuring the expression levels of thousands of genes per cell, single-cell RNA sequencing (scRNA-seq) enables an unbiased classification of cell types and fine characterization of functional cell states (1, 2). The bioinformatics analyses of scRNA-seq datasets allow the identification of new cell types and functional subsets (3), and may reconstruct cellular differentiation or activation dynamics *a posteriori* from snapshot data (4–7).

All scRNA-seq protocols are based on four common steps: (i) single cell isolation, (ii) reverse transcription (RT) of mRNA, (iii) amplification of cDNA, and (iv) preparation of next-generation sequencing libraries. Single cell isolation can be performed through FACS or nanodroplet encapsulation. FACS has the advantage of allowing the user to record the precise cell surface phenotype of each sorted cell (index sorting) and link it to its deeply sequenced transcriptome (>2,000 genes/cell), but with a limited throughput of a few hundred cells per sample (8, 9). Nanodroplet encapsulation, as performed in the commercial system proposed by 10× Genomics (10), enables an easy parallel processing of thousands of single cells, albeit at the cost of reduced sensitivity (around 1,000 genes/cell in peripheral blood lymphocytes). Depending on the protocols, the scRNA-seq reads which are used for gene expression analysis may cover the whole mRNA sequence (full-length sequencing) or only the beginning or the end of mRNA molecules (5′-end or 3′-end sequencing, respectively). Because every scRNA-seq protocol has its own strengths and limitations (11, 12), method choice should be driven by the biological issue at hand but will also be constrained by the desired depth (number of genes detected per cell), throughput (number of cells analyzed per sample), and budget.

In the adaptive immune system, complex gene rearrangements generate diverse B cell receptor (BCR) and T cell receptor (TCR) repertoires, enabling the recognition of an infinite range of antigens by B and T cells, respectively. When stimulated by their cognate antigen, each B or T cell clone can differentiate into multiple effector cell types that differ transcriptionally and functionally (13, 14). In the process of differentiation, the TCR sequence of mature T cells remains unchanged, while the BCR sequence of B cells may be altered in affinity maturation events such as class switch recombination and somatic hypermutation (15). The progeny of a single T or B cell can thus be accurately identified through identical (for TCR) or very similar (for BCR) V–J junctional sequences in their TCR or BCR chain genes, respectively. Integrating single-cell immunoglobulin heavy chain (IGH) sequencing with low-throughput gene expression analysis by single-cell qPCR already revealed important features of memory B cell diversification (16) and B cell lymphoma evolution (17). Methods which enable the parallel analysis of repertoire sequence and whole transcriptome gene expression in single B or T cells are required to deeply investigate the multiple aspects of lymphocyte biology and malignancy.

Experimental and computational approaches to infer TCR and BCR sequence from scRNA-seq datasets of T and B cells have been developed, relying either on data produced by plate-based full-length sequencing (Smart-seq2) (18–22) or droplet-based 5′-end sequencing (10× Genomics) (23). The former allows for a deep analysis of phenotypically defined FACS-sorted cells but is costly, labor intensive, and does not support unique molecular identifiers (UMIs, random DNA barcodes incorporated into cDNA molecules during RT and enabling the quantitative deconvolution of scRNA-seq reads despite potential cDNA amplification artifacts). The latter incorporates UMIs, is relatively cheap, and is easy to perform on thousands of cells but does not allow the precise selection of phenotypically defined cells and requires the production and sequencing of additional libraries for BCR or TCR sequencing.

Here we present FB5P-seq, a novel protocol for 5′-end scRNA-seq analysis of FACS-sorted cells, which incorporates UMI for accurate molecular counting and allows direct efficient recovery of paired BCR and TCR repertoire sequences when applied to B and T cells. We report the good sensitivity and accuracy of FB5P-seq when applied to human tonsil B cell subsets and antigen-specific peripheral blood T cells, highlighting the relevance and performance of our cost-effective and scalable technology.

## Results

### FB5P-seq Experimental Workflow

**Figure 1:**
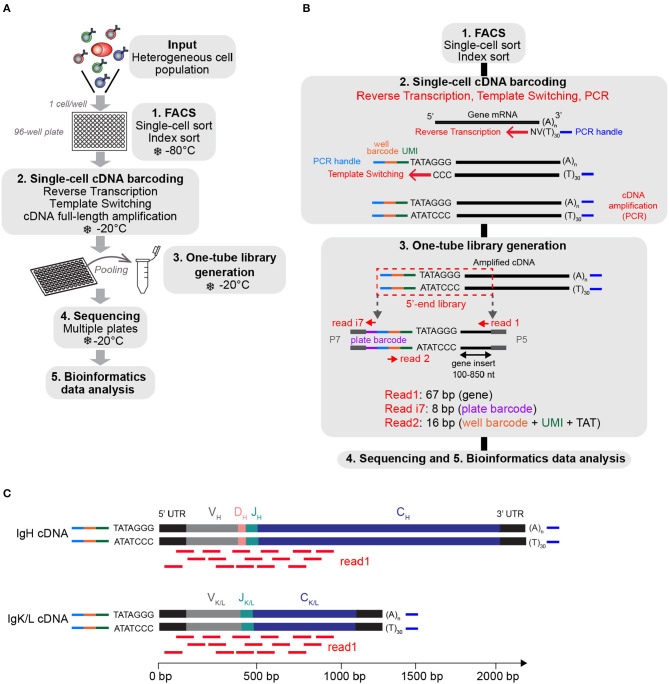
Overview of the FB5P-seq experimental workflow. **(A)** Major experimental steps of the FB5P-seq workflow. **(B)** Schematic overview of the molecular designs and the reactions in the FB5P-seq workflow. **(C)** Schematic illustration of the mapping of the Read1 sequences (in red) on IGH and IGK or IGL amplified cDNA, enabling the *in silico* reconstruction of paired variable BCR sequences.

We based the design of the FB5P-seq experimental workflow on existing full-length (8) and 5′-end (9, 24) scRNA-seq protocols. The main originalities in FB5P-seq were to perform cell-specific barcoding, incorporate 5-bp UMI during reverse transcription, and sequence the 5′-ends of amplified cDNAs from their 3′-end, not from the transcription start site (Figures 1A,B). In FB5P-seq, single cells of interest are sorted in 96-well plates by FACS, routinely using a 10-color staining strategy to identify and enrich specific subsets of B or T cells while recording all parameters through index sorting. Single cells are collected in lysis buffer containing External RNA Controls Consortium (ERCC) spike-in mRNA (0.025 pg per well), and the sorted plates are immediately frozen on dry ice and stored at −80°C until further processing. ERCC controls are a mix of 92 distinct synthetic polyadenylated RNAs of distinct length with known sequences and concentrations which cover a broad range of abundance levels. ERCC controls are reverse-transcribed and amplified along endogenous mRNA and may therefore be used to control the accuracy and molecular sensitivity of scRNA-seq methods (see section Materials and Methods) (11, 12, 24). The amount of ERCC spike-in mRNA added to each well was optimized to yield around 5% of sequencing reads covering ERCC genes when studying lymphocytes which generally contain little mRNA. mRNA reverse transcription, cDNA 5′-end barcoding, and PCR amplification are performed with a template switching (TS) approach. Notably, our TSO design included a PCR handle (different from the one introduced at the 3′-end upon RT priming), an 8-bp well-specific barcode followed by a 5-bp UMI, a TATA spacer (25), and three riboguanines. We empirically selected the 96 well-specific barcode sequences to avoid TSO concatemers in FB5P-seq libraries. After amplification, the barcoded full-length cDNAs from each well are pooled for purification and one-tube library preparation. For each plate, an Illumina sequencing library targeting the 5′-end of barcoded cDNA is prepared by a modified transposase-based method (tagmentation, a process where full-length cDNA is fragmented into small fragments by a Tn5 transposase enzyme which incorporates specific adapter sequences at the ends of the processed DNA strands), incorporating a plate-associated i7 barcode. The FB5P-seq library preparation protocol is cost-effective (260 € for the library preparation of a 96-well plate), is easily scalable, and may be implemented on a pipetting robot.

FB5P-seq libraries are sequenced in paired-end single-index mode (i.e., sequencing both ends of the sequencing libraries and one library-specific barcode index, a standard Illumina sequencing protocol) with Read1 covering the gene insert from its 3′-end, Read i7 assigning the plate barcode, and Read2 covering the well-barcode and UMI. Because FB5P-seq libraries have a broad size distribution, with a gene insert of 100–850 bp, Read 1 sequences cover the 5′-end of transcripts approximately from 30 to 850 bases downstream of the transcription start site. Consequently, sequencing reads cover the whole variable and a significant portion of the constant region of the *IGH*- and *IGK/L*-expressed mRNAs (Figure 1C), enabling *in silico* assembly and reconstitution of BCR repertoire from scRNA-seq data. Because TCRα and TCRβ genes share a similar structure, FB5P-seq is equally suitable for reconstructing TCR repertoire from scRNA-seq data when T cells are analyzed.

### FB5P-seq Bioinformatics Workflow

**Figure 2:**
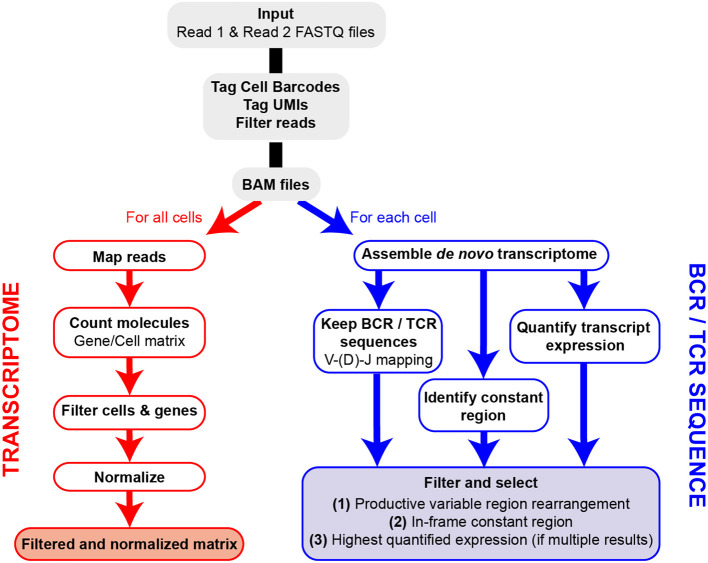
Overview of FB5P-seq bioinformatics workflow. Major steps of the bioinformatics pipeline starting from Read1 and Read2 FASTQ files for the generation of single-cell gene expression matrices and BCR or TCR repertoire sequences.

The FB5P-seq data are processed to generate both a single-cell gene count matrix and single-cell BCR or TCR repertoire sequences when analyzing B cells or T cells, respectively. After extracting the well-specific barcode and UMI from Read2 sequences and filtering out low-quality or unassigned reads, we use two separate pipelines for gene expression and repertoire analysis (Figure 2). The transcriptome analysis pipeline was derived from the Drop-seq pipeline (26). Briefly, it consists of mapping all Read1 sequences to the reference genome, then quantifying, for each gene in each cell, the number of unique molecules through UMI sequences. After merging the data from all 96-well plates in the experiment, we filter the resulting gene-by-cell count matrices to exclude low-quality cells and normalize by total UMI content per cell (see section Materials and Methods).

For the extraction of BCR or TCR repertoire sequences from FB5P-seq data, we have developed our own pipeline based on *de novo* single-cell transcriptome assembly and mapping of reconstituted long transcripts (contigs or isoforms) on public databases of variable immunoglobulin or TCR genes. We identify and select contigs corresponding to productive V(D)J rearrangements in-frame with an identified constant region gene. In cases where multiple isoforms are identified for a given chain (e.g., *IGH*) in a single cell, we assign the most highly expressed isoform and discard the other one(s). In early validation experiments, our pipeline was equally efficient and accurate as RT-PCR followed by Sanger sequencing for *IGH* variable region analysis (data not shown), with the major advantage of retrieving complete variable regions and large portions of constant regions of both *IGH* and *IGK/L*, or *TCRA* and *TCRB*, from the same scRNA-seq run.

### FB5P-seq Quality Metrics on Human Tonsil B Cell Subsets

**Figure 3:**
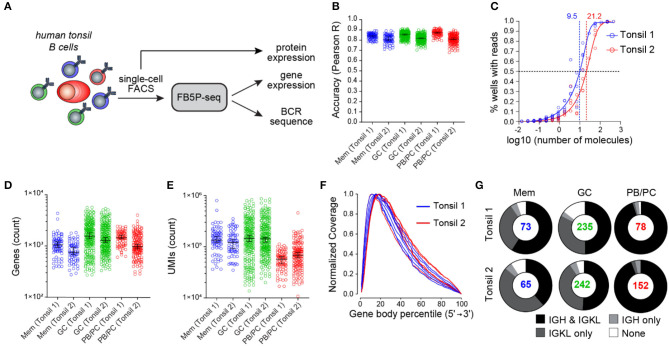
FB5P-seq quality metrics on human tonsil B cell subsets. **(A)** Experimental workflow for studying the human tonsil B cell subsets with FB5P-seq. **(B)** Per-cell quantitative accuracy of FB5P-seq computed based on ERCC spike-in mRNA detection (see section Materials and Methods) for memory B cells (Mem, *n* = 73 tonsil 1, *n* = 65 tonsil 2), GC B cells (GC, *n* = 235 tonsil 1, *n* = 242 tonsil 2), and PB/PCs (*n* = 78 tonsil 1, *n* = 152 tonsil 2). The black line indicates the median with 95% confidence interval error bars. **(C)** Molecular sensitivity of FB5P-seq computed on ERCC spike-in mRNA detection rates (see section Materials and Methods) in two distinct experiments. The dashed lines indicate the number of ERCC molecules required to reach a 50% detection probability. **(D,E)** Total number of unique genes **(D)** and molecules **(E)** detected in human tonsil Mem B cells (*n* = 73 tonsil 1, *n* = 65 tonsil 2), GC B cells (*n* = 235 tonsil 1, *n* = 242 tonsil 2), and PB/PCs (*n* = 78 tonsil 1, *n* = 152 tonsil 2). The black line indicates the median with 95% confidence interval error bars. **(F)** Gene body coverage analysis for tonsil 1 (*n* = 5) and tonsil 2 (*n* = 6) plate libraries. Each curve was computed from the BAM file corresponding to one library from a 96-well plate pool (see section Materials and Methods). **(G)** Pie charts showing the relative proportion of cells with reconstructed productive IGH and IGK/L sequences (black), only IGK/L sequences (dark gray), only IGH sequences (light gray), or no BCR sequence (white) among Mem B cells, GC B cells, and PB/PCs from tonsil 1 and tonsil 2 samples. The total number of cells analyzed for each subset is indicated at the center of the pie chart.

To illustrate the performance of our scRNA-seq protocol, we obtained non-malignant tonsil cell suspensions from two adult human donors, referred to as Tonsil 1 and Tonsil 2. Based on surface marker staining, we excluded monocytes, T cells, and naïve B cells and sorted memory (Mem) B cells, germinal center (GC) B cells, and plasmablasts or plasma cells (PB/PCs) for FB5P-seq analysis (Figure 3A). We processed the Tonsil 1 and Tonsil 2 samples in two separate experiments, generating libraries from five and six plates, respectively. The libraries were sequenced at an average depth of ~500,000 reads per cell (Table S1). After bioinformatics quality controls (see section Materials and Methods), we retained more than 90% of cells in the gene expression dataset (Table S1). We computed per-cell accuracy (Figure 3B) and per-experiment sensitivity (Figure 3C) based on ERCC spike-in detection levels and rates, respectively (11, 12). All cells showed high quantitative accuracy independently of their phenotype, with an overall mean correlation coefficient of 0.83 (Figure 3B). The molecular sensitivity ranged from 9.5 to 21.2 (Figure 3C), which compares favorably with other current scRNA-seq protocols (11, 12). We detected a mean of 987, 1,712, and 1,307 genes per cell in Mem B cells, GC B cells, and PB/PCs, respectively (Figure 3D). The GC and Mem B cells displayed higher total molecule counts (mean UMI counts of 192,765 and 145,356, respectively) than the PB/PCs (mean UMI count of 67,861) (Figure 3E).

As expected from the method design, the FB5P-seq Read1 sequence coverage was biased toward the 5′-end of gene bodies, with a broad distribution robustly covering from the 3rd to the 60th percentile of the gene body length on average (Figure 3F). In Tonsil 1 and Tonsil 2 B cell subsets, the BCR reconstruction pipeline retrieved at least one productive BCR chain for the majority of the cells (Figure 3G). Consistent with the high expression of BCR gene transcripts for sustained antibody production, we obtained the paired *IGH* and *IGK/L* repertoire for the vast majority of PB/PCs. In Mem and GC B cells, we obtained paired *IGH* and *IGK/L* sequences on ~50% of the cells and only the *IGK/L* sequence in most of the remaining cells. The superior recovery of *IGK/L* sequences was likely because the expression level of *IGK/L* was about two-fold higher than that of the *IGH* in our FB5P-seq data (data not shown).

Altogether accuracy, sensitivity, gene coverage, and BCR sequence recovery highlighted the high performance of the FB5P-seq method for the integrative analysis of transcriptome and BCR repertoire in single B cells.

### FB5P-seq Analysis of Human Tonsil B Cell Subsets

**Figure 4:**
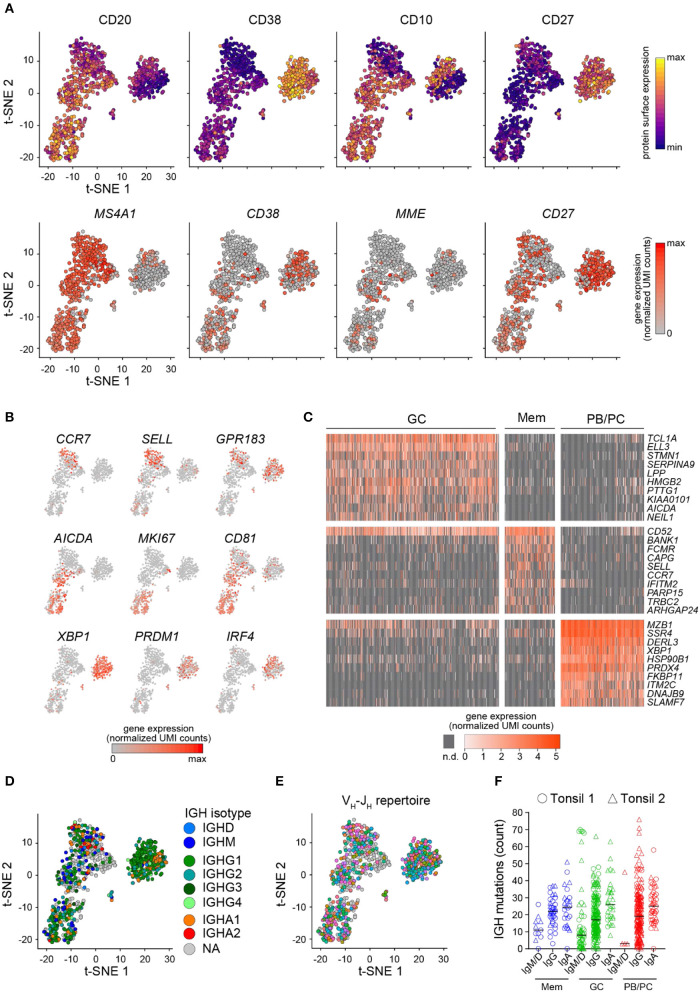
FB5P-seq analysis of human tonsil B cell subsets. **(A)** t-SNE map of single human tonsil B cell subsets computed on 4,000 variable genes, excluding the BCR genes. The cells are colored based on the surface protein (upper panel) or the corresponding gene (lower panel) expression of the indicated markers (*n* = 845 cells). **(B)** Expression of the indicated marker genes for Mem B cells (upper panel), GC B cells (middle panel), or PB/PCs (bottom panel*)* in single human tonsil B cells laid out in the t-SNE map. **(C)** Gene expression heatmap of GC B cells (*n* = 477), Mem B cells (*n* = 138), and PB/PCs (*n* = 230) for the top 10 marker genes of each subset (n.d.: not detected). **(D,E)** t-SNE map of single human tonsil B cell subsets colored by IGH isotype (NA: not applicable, i.e., no IGH reconstructed) **(D)** or V_H_-J_H_ repertoire (gray cells: no IGH reconstructed) **(E)**. **(F)** Scatter plots showing the IGH mutation frequency in human tonsil 1 (circles) and tonsil 2 (triangles) B cells sorted by their IGH isotype and phenotype (Mem B cells: *n* = 11 IgM/IgD+, *n* = 37 IgG+, and *n* = 26 IgA+; GC B cells: *n* = 55 IgM/IgD+, *n* = 174 IgG+, and *n* = 32 IgA+; PB/PCs: *n* = 4 IgM/IgD+, *n* = 179 IgG+, and *n* = 42 IgA+ PB/PCs. The black line indicates the median.

As a biological proof-of-concept, we further analyzed the Tonsil 1 and Tonsil 2 datasets. T-distributed stochastic neighbor embedding (t-SNE) analysis on the gene expression data discriminated three major cell clusters. Tonsil B cells clustered based on their sorting phenotype (Mem B cells, GC B cells, or PB/PCs) and did not cluster by sample origin (Figure S1A). Cell cycle status further separated the cycling (S and G2/M phase) from the non-cycling (G1) GC B cells (Figure S1B). The expression levels of surface protein markers recorded through index sorting were consistent with the gating strategy of Mem B cells (CD20^+^CD38^lo^CD10^−^CD27^+^), GC B cells (CD20^+^CD38^+^CD10^+^), and PB/PCs (CD38^hi^CD27^hi^) (Figure 4A, top panel). The expression of the corresponding mRNAs mirrored the protein expression (Figure 4A, bottom panel) but revealed numerous cells where the mRNA was undetected despite the intermediate or high levels of the protein. Further, we detected the expression of known marker genes for Mem B cells (*CCR7, SELL*, and *GPR183*), GC B cells (*AICDA, MKI67*, and *CD81*), or PB/PCs (*XBP1, PRDM1*, and *IRF4*) in the corresponding clusters (Figure 4B) and identified the top marker genes for each cell subset (Figure 4C). Those analyses were consistent with previous single-cell qPCR analyses (17) and bulk microarray analyses of human B cell subsets (27, 28).

Integrating the single-cell BCR repertoire data to the t-SNE embedding, we revealed that the *IGH* and *IGK/L* repertoire of tonsil B cell subsets was polyclonal (Figures 4D,E and Figures S1C,D). Interestingly, while the somatic mutation load was equivalent in Igκ and Igλ light chains from Mem B cells, GC B cells, and PB/PCs (Figure S1E), the *IGH* mutation rate depended on isotype, with the IgA^+^ cells expressing the most mutated BCR (Figure 4F) regardless of phenotype or sample origin. By contrast, the IgM/IgD^+^ cells exhibited the lowest somatic mutation loads (Figure 4F).

Overall, those analyses confirmed that the FB5P-seq method is relevant for simultaneous protein, whole-transcriptome, and BCR sequence analysis in human B cells.

### FB5P-seq Analysis of Human Peripheral Blood Antigen-Specific CD4 T Cells

**Figure 5:**
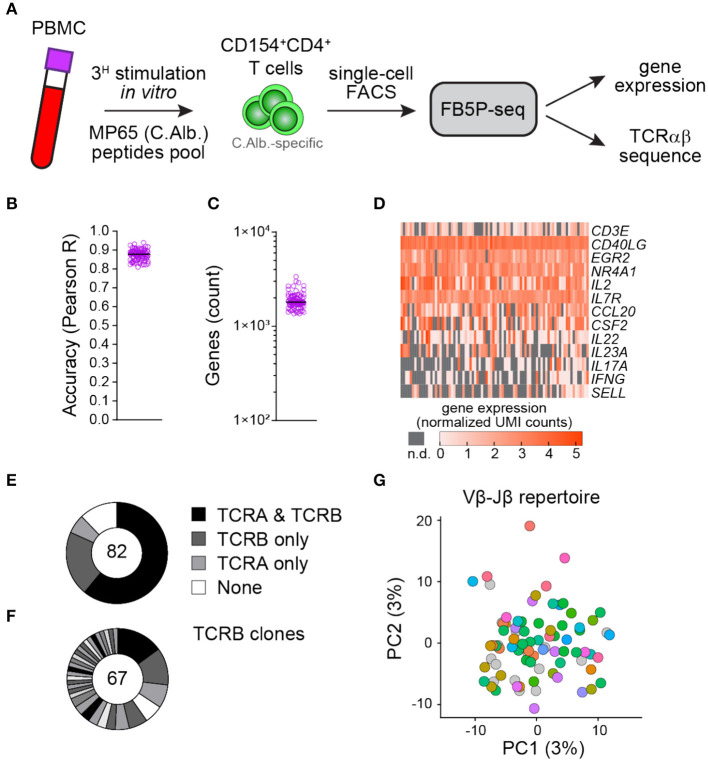
FB5P-seq analysis of the human peripheral blood antigen-specific CD4 T cells. **(A)** Experimental workflow for studying the human peripheral blood *C. albicans*-specific CD4 T cells with FB5P-seq. **(B)** Per-cell quantitative accuracy of FB5P-seq computed based on ERCC spike-in mRNA detection (see section Materials and Methods) for *C. albicans*-specific CD4 T cells (*n* = 82). The black line indicates the median with 95% confidence interval error bars. **(C)** Total number of unique genes detected in *C. albicans*-specific CD4 T cells (*n* = 82). The black line indicates the median with 95% confidence interval error bars. **(D)** Gene expression heatmap of human peripheral blood *C. albicans*-specific CD4 T cells for a selected panel of marker genes (n.d.: not detected). **(E)** Pie charts showing the relative proportion of cells with reconstructed productive TCRA and TCRB sequences (black), only TCRB sequences (dark gray), only TCRA sequences (light gray), or no TCR sequence (white) among *C. albicans*-specific CD4 T cells (*n* = 82). **(F)** Distribution of TCRB clones among *C. albicans*-specific CD4 T cells (*n* = 67). The black and gray sectors indicate the proportion of TCRB clones (clonotype expressed by at least two cells) within the single cells analyzed (white sector: unique clonotypes). **(G)** Projection of *C. albicans*-specific CD4 T cells (*n* = 82) on the first two PCs computed on 4,000 variable genes, excluding the TCR genes (PC1: 3% of total variability, PC2: 3% of total variability). The cells are colored based on the V_β_-J_β_ repertoire (gray cells: no TCRB reconstructed).

To test whether our protocol is also effective in T cells, we applied FB5P-seq to *Candida albicans*-specific human CD4 T cells sorted after a brief restimulation of fresh peripheral blood mononuclear cells with a pool of MP65 antigen-derived peptides (29) (Figure 5A and section Materials and Methods). *C. albicans* is a common commensal in humans known to generate antigen-specific circulating memory CD4 T cells with a T_H_17 profile (30). Similar to the B cell dataset, the T cell dataset displayed high per-cell accuracy (Figure 5B) and an average of 1,890 detected genes per cell (Figure 5C). Gene expression analysis showed an efficient detection of T cell marker genes (*CD3E*), activation genes (*CD40LG, EGR2, NR4A1*, and *IL2*), and TH17-specific genes (*CCL20, CSF2, IL22, IL23A*, and *IL17A*) in those reactivated antigen-specific T cells (Figure 5D). We recovered at least one productive TCRα or TCRβ chain in 88% of cells and paired TCRαβ repertoire in 61% of cells (Figure 5E). Moreover, the CDR3β sequence analysis revealed some expanded TCRβ clonotypes likely related to MP65 antigen specificity (Figure 5F). A principal component analysis of the gene expression data and the visualization of V_β_-J_β_ TCR rearrangements revealed no apparent segregation of antigen-specific T cells expressing different clonotypes (Figure 5G).

Taken together, these data indicate that our method is also relevant for integrative scRNA-seq analysis of human T cells, especially for tracking rare antigen-specific cells *ex vivo*.

## Discussion

FB5P-seq is a novel 5′-end scRNA-seq workflow that allows an accurate gene expression analysis of hundreds of FACS-sorted cells in parallel. When applied to B or T cells, the FB5P-seq data support the *in silico* reconstruction of paired full-length BCR or TCR variable repertoire on a per-cell basis. As demonstrated by our studies of human tonsil B cells and peripheral blood antigen-specific T cells, FB5P-seq is particularly adapted for the integrative analysis of surface phenotype (through index sorting), gene expression, and antigen receptor repertoire of rare phenotypically defined B or T cells. Because FB5P-seq integrates three layers of barcodes (UMIs, cellular barcodes, and plate barcodes), the workflow is more cost- and time-effective than Smart-Seq2 (31), which has been so far the method of choice for plate-based scRNA-seq analysis of gene expression and antigen receptor repertoire in B or T cells (18, 19, 21, 22). The molecular detection limit of FB5P-seq (10–20 molecules) was higher than what has been reported for Smart-seq2 (11) (seven molecules), suggesting that FB5P-seq may recover the expression of less genes per cell. In a recent benchmarking study of scRNA-seq methods, Smart-Seq2 detected the expression of ~2,500 genes per cell in human peripheral blood mononuclear cells (PBMCs) at a sequencing depth of 1 × 10^6^ reads per cell (32). The sensitivity of FB5P-seq was sufficient to recover in the order of 1,000–2,000 genes per cell on average in human lymphocytes (depending on cell type and cell cycle status) at a sequencing depth of ~5 × 10^5^ reads per cell.

One of our main constraints when developing FB5P-seq was to enable the *in silico* reconstruction of BCR or TCR repertoire sequences directly from the scRNA-seq reads. Because the variable regions of antigen receptor genes are encoded from the 5′-end of mRNAs, 3′-end scRNA-seq protocols are not suitable for the parallel analysis of BCR or TCR repertoire, unless a separate amplification with cell-specific and gene-specific primers is performed on full-length cDNA before pooling single-cell contents (33). Full-length scRNA-seq protocols with paired-end sequencing have been used successfully for parallel transcriptome and repertoire sequencing of single B and T cells, with dedicated bioinformatics pipelines named TraCeR (18), BASIC (22), BraCeR (19), and VDJPuzzle (21). In FB5P-seq, the sequencing reads mainly cover the 5′-ends of mRNAs in a 3′ to 5′ orientation, which generates a broad coverage of the most 5′ half of mRNAs when using 67-nucleotide-long reads. As a consequence, FB5P-seq can successfully reconstruct BCR or TCR repertoire sequences from the scRNA-seq reads when sequencing libraries on a 75-cycle Illumina flow cell at a targeted read depth of 5 × 10^5^ reads per cell, as had been done for the datasets presented here. In that configuration, the cost of performing FB5P-seq is roughly 5 € per cell (2.5 € library preparation + 2.5 € sequencing), which is significantly lower than Smart-Seq2 (30 € library preparation + 2.5–5 € sequencing) (11). In FB5P-seq, only one library preparation is required per 96-well plate, which drastically reduces the handling time when working with manual pipetting, as compared to generating one library per cell in Smart-Seq2. Yet because library preparation only requires a small amount of pooled cDNA, the full-length amplified cDNA from each single-cell can be archived and used for downstream applications such as single-cell antibody cloning (34).

Our bioinformatics pipeline for recovering repertoire sequences differs from previously published solutions (18, 19, 21, 22) in that it performs *de novo* transcriptome reconstruction on all sequences prior to filtering contigs corresponding to the rearranged BCR or TCR chains. Because competing algorithms rely on full-length scRNA-seq paired-end reads, we did not test their ability to recover BCR or TCR sequences on FB5P-seq reads. Yet the demonstrated performance of our method on human B cell subsets, with close to 100% paired heavy and light chain reconstruction efficiency on PB/PCs, suggests that it performs at least equally well as those methods.

We have designed the FB5P-seq TSOs with short five-nucleotide UMIs, enabling the detection of a maximal molecular diversity of 4^5^ = 1,024 molecules for each gene per cell. Although this may be low for some genes in some cell types, in our analyses on lymphocytes, only the *CD74* gene showed recurrent saturating levels in Mem B cells and GC B cells (not shown). Longer UMI sequences could be used in the TSO design to prevent saturation issues (10, 24), but template switching efficiency may be affected by increased TSO length (35). In our analyses of human lymphocytes, we have used very low levels of ERCC spike-ins (0.025 pg per well, corresponding to a 1:2,000,000 final dilution) compared to those of other scRNA-seq protocols (11, 12, 36). We have optimized the concentration of ERCC spike-ins for having <5% of sequencing reads mapping on ERCC genes to maximize the amount of biological information in our experiments while retaining the usefulness of ERCC spike-ins for quality control purposes. This would need to be adjusted when studying cell types with more mRNA content than the primary lymphocytes.

The FB5P-seq protocol was designed to use FACS single cell sorting for cell deposition into 96-well plates. Most cell sorting machines and software enable the use of index sorting to record the flow cytometry parameters (cell size and fluorescence levels for each channel) of each sorted cell, including for markers that were not used in the gating strategy but were included during cell staining. This may be used to analyze mRNA/protein levels in parallel or to investigate the relevance of cell surface markers to delineate transcriptionally robust cell subsets (7). In our implementation of FB5P-seq, we use the 96-well plate format, sort single cells into 2-μl lysis buffer, and use manual pipetting throughout the protocol, but our method would only require minor adjustments to work on 384-well plates with smaller volumes and automatic liquid handlers like the MARS-seq protocol (37, 38). Furthermore, plates containing the lysis buffer can be stored at −80°C and shipped on dry ice, before and after cell sorting, facilitating collaborative work and multisite projects.

High throughput droplet-based 5′-end scRNA-seq (10× Genomics 5′-end) is an excellent option to analyze a transcriptome and repertoire sequence for thousands of cells in parallel in complex tissues (23, 39). Combining 5′-end scRNA-seq with DNA-tagged antibody labeling and barcoding may further enable the multiplexing of several samples and the analysis of the surface phenotype of cells (40). Yet, because those approaches best apply when many thousands of cells are available for input, we believe that FB5P-seq is a valuable option to obtain the same multimodal information when focusing on rare cells defined by a complex surface phenotype. Furthermore, high-throughput methods may be less appropriate when one of the protein markers to be tracked in the analyzed cells is an intracellular fluorescent reporter (e.g., lineage tracer, biochemical indicator) which will not be accessible with DNA-tagged antibody labeling in intact live cells. Another limitation of high throughput droplet-based 5′-end scRNA-seq is the difficulty to recover BCR sequences from non-antibody producing cells (e.g., naïve, Mem, or GC B cells, expressing low to moderate amounts of *IGH* and *IGK/L* transcripts) when antibody-producing cells (PB/PCs, expressing on the order of 100-fold more *IGH* and *IGK/L* transcripts than other B cells) are present in the analyzed sample. In that situation, most of the molecules in the BCR sequencing library will be coming from PB/PCs, leaving too few reads to successfully reconstruct a BCR repertoire in the other B cells (our unpublished observations and https://support.10xgenomics.com/single-cell-vdj/software/overview/design). In FB5P-seq, the targeted sequencing depth (5 × 10^5^ reads per cell) is sufficient to reconstruct the BCR repertoire for all B cell types, and the efficiency of our method is not dependent on the proportion of PB/PCs in the sample.

Thus, FB5P-seq is a novel alternative to Smart-seq2 and 10× Genomics 5′-end scRNA-seq for the integrative analysis of transcriptome and antigen receptor repertoire in B and T cells (Table 1). We expect that our method will provide valuable insights for decoding the mechanisms regulating the molecular and functional diversity of lymphocytes during protective immunity, auto-immunity, cancer immunity, or lymphoma development and progression.

**Table 1:** Key methodological features of FB5P-seq, Smart-seq2, and 10x Genomics 5′-end integrative scRNA-seq methods.

	**FB5P-seq**	**Smart-seq2**	**10× Genomics 5^**′**^-end**
Cell capture	FACS-based	FACS-based	Droplet-based
Analysis of phenotypic markers	From FACS staining and index sorting May include fluorescent reporters or indicators	From FACS staining and index sorting May include fluorescent reporters or indicators	From DNA-barcoded antibody stainings (e.g., CITE-seq) Limited to surface markers
Cell throughput	One cell/well in 96-well plate format Multiplex up to 16 plates per sample (our experience)	One cell/well in 96-well plate format Multiple plates may be multiplexed	Up to 10,000 cells per run Multiple runs may be done in parallel
cDNA barcoding	RT with template switching 5′-end barcoding with UMI	RT with template switching No cDNA barcoding, no UMI	RT with template switching 5′-end barcoding with UMI
Sequencing library	One library per 96-well plate	One library per cell	One library per run for gene expression One library per run for BCR One library per run for TCR One library per run for surface markers
Recommended sequencing depth	5 × 10^5^ reads/cell Paired-end, single-index	1 × 10^6^ reads/cell Single-end or paired-end, dual-index	5 × 10^4^ reads/cell for gene expression 5 × 10^3^ reads/cell for BCR/TCR 5 × 10^3^ reads/cell for surface markers Paired-end, single-index
Sensitivity (for PBMC, B/T cells)	1,000–2,000 genes/cell	2,000–3,000 genes/cell	1,000–2,000 genes/cell

## Materials and Methods

### Human Samples

Non-malignant tonsil samples from a 35-year-old male (Tonsil 1) and a 30-year-old female (Tonsil 2) were obtained as frozen live cell suspensions from the CeVi collection of the Institute Carnot/Calym (ANR, France, https://www.calym.org/-Viable-cell-collection-CeVi-.html).

Peripheral blood mononuclear cells were collected in Nantes University Hospital and used fresh in the peptide restimulation assays for isolating *C. albicans*-specific T cells.

Written informed consent was obtained from the donors.

### Flow Cytometry and Cell Sorting of B Cell Subsets

Frozen live cell suspensions were thawed at 37°C in RPMI + 10% FCS and then washed and resuspended in FACS buffer [phosphate-buffered saline (PBS) + 5% FCS + 2 mM EDTA] at a concentration of 10^8^ cells/ml for staining. The cells were first incubated with 2% normal mouse serum and Fc-Block (BD Biosciences) for 10 min on ice. Then, the cells were incubated with a mix of fluorophore-conjugated antibodies (Table S2) for 30 min on ice. The cells were washed in PBS and then incubated with the Live/Dead Fixable Aqua Dead Cell Stain (Thermofisher) for 10 min on ice. After a final wash in FACS buffer, the cells were resuspended in FACS buffer at a concentration of 10^7^ cells/ml for cell sorting on a four-laser BD FACS Influx (BD Biosciences).

Mem B cells were gated as CD3^−^CD14^−^IgD^−^CD20^+^CD10^−^CD38^lo^CD27^+^SSC^lo^single live cells. GC B cells were gated as CD3^−^CD14^−^IgD^−^CD20^+^CD10^+^CD38^+^ single live cells. PB/PCs were gated as CD3^−^CD14^−^IgD^−^CD38^hi^CD27^+^SSC^hi^ single live cells.

### Restimulation and Cell Sorting of Antigen-Specific T Cells

Fresh PBMCs (10–20 × 10^6^ cells, final concentration 10 × 10^6^ cells/ml) were stimulated for 3 h at 37°C with 0.6 nmol/ml PepTivator *C. albicans* MP65 (pool of 15-amino-acid-length peptides with 11 amino acid overlap, Miltenyi Biotec) in RPMI + 5% human serum in the presence of 1 μg/ml anti-CD40 (HB14, Miltenyi Biotec). After stimulation, the PBMCs were labeled with PE-conjugated anti-CD154 (5C8, Miltenyi Biotec) and enriched with anti-PE magnetic beads (Miltenyi Biotec) (41). After enrichment, the cells were stained with PerCP-Cy5.5 anti-CD4 (RPA-T4, Biolegend), AlexaFluor700 anti-CD3 (SK7, Biolegend), and APC-Cy7 anti-CD45RA (HI100, Biolegend), and the antigen-specific T cells were gated as CD3^+^CD4^+^CD45RA^−^CD154^+^ single live cells for single cell sorting.

### Single-Cell RNA-seq

Single cells were FACS-sorted into ice-cold 96-well PCR plates (Thermofisher) containing 2 μl lysis mix per well. The lysis mix contained 0.5 μl 0.4% (v/v) Triton X-100 (Sigma-Aldrich), 0.05 μl 40 U/μl RnaseOUT (Thermofisher), 0.08 μl 25 mM dNTP mix (Thermofisher), 0.5 μl 10 μM (dT)30_Smarter primer (8) (Table S3), 0.05 μl 0.5 pg/μl External RNA Controls Consortium (ERCC) spike-ins mix (Thermofisher), and 0.82 μl PCR-grade H_2_0 (Qiagen).

For B cell subset sorting, the index sorting mode was activated to record the different fluorescence intensity of each sorted single cell. Index-sorting FCS files were visualized in FlowJo software, and compensated parameter values were exported in CSV tables for further processing. For visualization on linear scales in the R programming software, we applied the hyperbolic arcsine transformation on fluorescence parameters. In every 96-well plate, two wells (H1 and H12) were left empty and processed throughout the protocol as negative controls.

Immediately after cell sorting, each plate was covered with an adhesive film (Thermofisher), briefly spun down in a benchtop plate centrifuge, and frozen on dry ice. The plates containing single cells in lysis mix were stored at −80°C and shipped on dry ice (only T cells) until further processing.

The plate containing the single cells in lysis mix was thawed on ice, briefly spun down in a benchtop plate centrifuge, and incubated in a thermal cycler for 3 min at 72°C (lid temperature 72°C). Immediately after, the plate was placed back on ice and 3 μl RT mastermix was added to each well. The RT mastermix contained 0.25 μl 200 U/μl SuperScript II (Thermofisher), 0.25 μl 40 U/μl RnaseOUT (Thermofisher), and 2.5 μl 2× RT mastermix. The 2× RT mastermix contained 1 μl 5× SuperScript II buffer (Thermofisher), 0.25 μl 100 mM DTT (Thermofisher), 1 μl 5 M betaine (Sigma-Aldrich), 0.03 μl 1 M MgCl_2_ (Sigma-Aldrich), 0.125 μl 100 μM well-specific template switching oligonucleotide TSO_BCx_UMI5_TATA (Tables S3, S4), and 0.095 μl PCR-grade H_2_O (Qiagen). Reverse transcription was performed in a thermal cycler (lid temperature 70°C) for 90 min at 42°C, followed by 10 cycles of 2 min at 50°C and 2 min at 42°C, and then 15 min at 70°C. The plates with single-cell cDNA were stored at −20°C until further processing.

For cDNA amplification, 7.5 μl LD-PCR mastermix was added to each well. The LD-PCR mastermix contained 6.25 μl 2× KAPA HiFi HotStart ReadyMix (Roche Diagnostics), 0.125 μl 20 μM PCR_Satija forward primer (9) (Table S3), 0.125 μl 20 μM SmarterR reverse primer (8) (Table S3), and 1 μl PCR-grade H_2_O (Qiagen). The amplification was performed in a thermal cycler (lid temperature 98°C) for 3 min at 98°C, followed by 22 cycles of 15 s at 98°C, 20 s at 67°C, 6 min at 72°C, and then a final elongation for 5 min at 72°C. The plates with amplified single-cell cDNA were stored at −20°C until further processing.

For library preparation, 5 μl of amplified cDNA from each well of a 96-well plate were pooled and completed to 500 μl with PCR-grade H_2_O (Qiagen). Two rounds of 0.6X solid-phase reversible immobilization beads (AmpureXP, Beckman, or CleanNGS, Proteigene) cleaning were used to purify 100 μl of pooled cDNA with final elution in 15 μl PCR-grade H_2_O (Qiagen). After quantification with Qubit dsDNA HS assay (Thermofisher), 800 pg purified cDNA pool was processed with the Nextera XT DNA sample Preparation kit (Illumina), according to the manufacturer's instructions with modifications to enrich the 5′-ends of tagmented cDNA during library PCR. After tagmentation and neutralization, 25 μl tagmented cDNA was amplified with 15 μl Nextera PCR Mastermix, 5 μl Nextera i5 primer (S5xx, Illumina), and 5 μl of a custom i7 primer mix (9) (0.5 μM i7_BCx + 10 μM i7_primer, Table S3). The amplification was performed in a thermal cycler (lid temperature 72°C) for 3 min at 72°C, 30 s at 95°C, followed by 12 cycles of 10 s at 95°C, 30 s at 55°C, 30 s at 72°C, and then a final elongation for 5 min at 72°C. The resulting library was purified with 0.8X solid-phase reversible immobilization beads (AmpureXP, Beckman, or CleanNGS, Proteigene).

Libraries generated from multiple 96-well plates of single cells and carrying distinct i7 barcodes were pooled for sequencing on an Illumina NextSeq550 platform, with High Output 75 cycles flow cells, targeting 5 × 10^5^ reads per cell in paired-end single-index mode with the following primers (Table S3) and cycles: Read1 (Read1_SP, 67 cycles), Read i7 (i7_SP, 8 cycles), and Read2 (Read2_SP, 16 cycles).

### Single-Cell RNA-seq Data Processing

We used a custom bioinformatics pipeline to process fastq files and generate single-cell gene expression matrices and BCR or TCR sequence files. Detailed instructions for running the FB5P-seq bioinformatics pipeline can be found at https://github.com/MilpiedLab/FB5P-seq. Briefly, the pipeline to obtain gene expression matrices was adapted from the Drop-seq pipeline (26), relied on extracting the cell barcode and UMI from Read2 and aligning Read1 on the reference genome using STAR and HTSeqCount. For BCR or TCR sequence reconstruction, we used Trinity for *de novo* transcriptome assembly for each cell based on Read1 sequences and then filtered the resulting isoforms for productive BCR or TCR sequences using MigMap, Blastn, and Kallisto. Briefly, MigMap was used to assess whether the reconstructed contigs corresponded to a productive V(D)J rearrangement and to identify germline V, D, and J genes and the CDR3 sequence for each contig. For each cell, the reconstructed contigs corresponding to the same V(D)J rearrangement were merged, keeping the largest sequence for further analysis. We used Blastn to align the reconstructed BCR or TCR contigs against the reference sequences of constant region genes and discarded the contigs with no constant region identified in-frame with the V(D)J rearrangement. Finally, we used the pseudoaligner Kallisto to map each cell's FB5P-seq Read1 sequences on its reconstructed contigs and quantify the contig expression. In cases where several contigs corresponding to the same BCR or TCR chain had passed the above filters, we retained the contig with the highest expression level.

The per well accuracy (Figure 3B) was computed as the Pearson correlation coefficient between log_10_(UMI_ERCC−xxxxx_ + 1) and log_10_(#mol_ERCC−xxxxx_ + 1), where UMI_ERCC−xxxxx_ is the UMI count for gene *ERCC-xxxxx* in the well and #mol_ERCC−xxxxx_ is the actual number of molecules for *ERCC-xxxxx* in the well (based on a 1:2,000,000 dilution in 2 μl lysis mix per well). For each well, only *ERCC-xxxxx* which were detected (UMI_ERCC−xxxxx_ > 0) were considered for calculating the accuracy.

To estimate sensitivity (Figure 3C), the percentage of wells with at least one molecule detected (UMI_ERCC−xxxxx_ > 0) was calculated over all the wells from five or six 96-well plates corresponding to human B cells sorted from Tonsil 1 or Tonsil 2, respectively. The value for each *ERCC-xxxxx* gene was plotted against log_10_(#mol_ERCC−xxxxx_). and a standard curve was interpolated with asymmetric sigmoidal 5PL model in GraphPad Prism 8.1.2 to compute the EC50 for each dataset.

The normalized coverage over genes (Figure 3F) was computed with RSeQC *geneBody_coverage* on *bam* files from 11 scRNA-seq 96-well plates corresponding to human B cells sorted from Tonsil 1 and Tonsil 2.

### Single-Cell Gene Expression Analysis

Quality control was performed on each dataset (Tonsil 1, Tonsil 2, and T cells) independently to remove poor-quality cells. Cells with <250 genes detected were removed. We further excluded cells with values below three median absolute deviations (MADs) from the median for UMI counts, for the number of genes detected, or for ERCC accuracy, and cells with values above three MADs from the median for ERCC transcript percentage.

For each cell, the gene expression UMI count values were log-normalized with Seurat v3 (42) *NormalizeData* with a scale factor of 10,000. Data from B cells of Tonsil 1 and Tonsil 2 were analyzed together. Data from *C. albicans*-specific T cells were analyzed separately. Four thousand variable genes, excluding BCR or TCR genes, were identified with Seurat *FindVariableFeatures*. After centering and scaling with Seurat *ScaleData*, principal component analysis was performed on variable genes with Seurat *RunPCA* and embedded in two-dimensional t-SNE plots with Seurat *RunT-SNE* on 40 principal components. Cell cycle phases were attributed with Seurat *CellCycleScoring* using lists of S phase- or G2/M phase-specific genes as described (43). Plots showing t-SNE embeddings colored by index sorting protein expression or other metadata (including BCR or TCR sequence-related information) were generated with ggplot2 *ggplot*. Plots showing t-SNE embeddings colored by gene expression were generated by Seurat *FeaturePlot*. Gene expression heatmaps were plotted with a custom function (available upon request).

For displaying the V–J repertoire polyclonality on t-SNE embeddings (Figures 4E, 5G), we used the VH and JH (or Vβ and Jβ for T cells) reference germline gene identities (identified by the MigMap algorithm, running IgBlast) to assign VH–JH clonotype or Vβ-Jβ clonotype (cells sharing the same VH and JH reference germline genes or sharing the same Vβ and Jβ reference germline genes, respectively) for each cell with a reconstructed IGH sequence (or TCRβ sequence, respectively). For display on the gene expression-based tSNE embedding, we used *ggplot* function, using the clonotype assignments as color-coded metadata.

## Accession Codes

The single-cell RNA-seq data generated in the current study are available in the Gene Expression Omnibus database under accession code GSE137275.

## Data Availability Statement

Detailed instructions for running the FB5P-seq bioinformatics pipeline can be found at https://github.com/MilpiedLab/FB5P-seq. The datasets generated for this study can be found in the NCBI Gene Expression Omnibus (GSE137275).

## Ethics Statement

Ethical review and approval was not required for the study on human participants in accordance with the local legislation and institutional requirements. The patients/participants provided their written informed consent to participate in this study.

## Author Contributions

NA developed the FB5P-seq, designed the experiments, performed the experiments, analyzed the data, and wrote the manuscript. IC-M developed the FB5P-seq, designed the bioinformatics pipeline, and analyzed the data. CD assembled and automatized the bioinformatics pipeline and analyzed the data. LG performed the experiments. AR performed the human T cell experiment. LS supervised the development and the assembly of the bioinformatics pipeline. PM developed the FB5P-seq, supervised the study, designed the experiments, analyzed the data, and wrote the manuscript. All authors reviewed and approved the manuscript.

### Conflict of Interest

NA, IC-M, LG, and PM are involved in a European Patent application that has been filed under no. EP19190782. The remaining authors declare that the research was conducted in the absence of any commercial or financial relationships that could be construed as a potential conflict of interest.

## References

[1] Giladi A Amit I. Immunology, one cell at a time. Nature. (2017) 547:27–9. 10.1038/547027a 28682352

[2] Linnarsson S Teichmann SA. Single-cell genomics: coming of age. Genome Biol. (2016) 17:97. 10.1186/s13059-016-0960-x 27160975 PMC4862185

[3] Villani A-C Satija R Reynolds G Sarkizova S Shekhar K Fletcher J. Single-cell RNA-seq reveals new types of human blood dendritic cells, monocytes, and progenitors. Science. (2017) 356:aah4573. 10.1126/science.aah4573 28428369 PMC5775029

[4] Trapnell C Cacchiarelli D Grimsby J Pokharel P Li S Morse M. The dynamics and regulators of cell fate decisions are revealed by pseudotemporal ordering of single cells. Nat Biotechnol. (2014) 32:381–6. 10.1038/nbt.2859 24658644 PMC4122333

[5] Tirosh I Venteicher AS Hebert C Escalante LE Patel AP Yizhak K. Single-cell RNA-seq supports a developmental hierarchy in human oligodendroglioma. Nature. (2016) 539:309–13. 10.1038/nature20123 27806376 PMC5465819

[6] Qiu X Mao Q Tang Y Wang L Chawla R Pliner HA. Reversed graph embedding resolves complex single-cell trajectories. Nat Methods. (2017) 14:979–82. 10.1038/nmeth.4402 28825705 PMC5764547

[7] Paul F Arkin Y Giladi A Jaitin DA Kenigsberg E Keren-Shaul H. Transcriptional heterogeneity and lineage commitment in myeloid progenitors. Cell. (2015) 163:1663–77. 10.1016/j.cell.2015.11.013 26627738

[8] Picelli S Faridani OR Björklund AK Winberg G Sagasser S Sandberg R. Full-length RNA-seq from single cells using Smart-seq2. Nat Protoc. (2014) 9:171–81. 10.1038/nprot.2014.006 24385147

[9] Satija R Farrell JA Gennert D Schier AF Regev A. Spatial reconstruction of single-cell gene expression data. Nat Biotechnol. (2015) 33:495–502. 10.1038/nbt.3192 25867923 PMC4430369

[10] Zheng GXY Terry JM Belgrader P Ryvkin P Bent ZW Wilson R. Massively parallel digital transcriptional profiling of single cells. Nat Commun. (2017) 8:14049. 10.1038/ncomms14049 28091601 PMC5241818

[11] Ziegenhain C Vieth B Parekh S Reinius B Guillaumet-Adkins A Smets M. Comparative analysis of single-cell RNA sequencing methods. Mol Cell. (2017) 65:631–43.e4. 10.1016/j.molcel.2017.01.023 28212749

[12] Svensson V Natarajan KN Ly L-H Miragaia RJ Labalette C Macaulay IC. Power analysis of single-cell RNA-sequencing experiments. Nat Meth. (2017) 14:381–7. 10.1038/nmeth.4220 28263961 PMC5376499

[13] Taylor JJ Pape KA Steach HR Jenkins MK Apoptosis and antigen affinity limits effector cell differentiation of a single naïve B cell. Science. (2015) 347:784–7. 10.1126/science.aaa1342 25636798 PMC4412594

[14] Tubo NJ Pagán AJ Taylor JJ Nelson RW Linehan JL Ertelt JM. Single naïve CD4+ T cells from a diverse repertoire produce different effector cell types during an infection. Cell. (2013) 153:785–96. 10.1016/j.cell.2013.04.007 23663778 PMC3766899

[15] McHeyzer-Williams M Okitsu S Wang N McHeyzer-Williams L. Molecular programming of B cell memory. Nat Rev Immunol. (2011) 12:24–34. 10.1038/nri3128 22158414 PMC3947622

[16] McHeyzer-Williams LJ Milpied PJ Okitsu SL McHeyzer-Williams MG. Class-switched memory B cells remodel BCRs within secondary germinal centers. Nat Immunol. (2015) 16:296–305. 10.1038/ni.3095 25642821 PMC4333102

[17] Milpied P Cervera-Marzal I Mollichella M-L Tesson B Brisou G Traverse-Glehen A. Human germinal center transcriptional programs are de-synchronized in B cell lymphoma. Nat Immunol. (2018) 19:1013. 10.1038/s41590-018-0181-4 30104629

[18] Stubbington MJT Lönnberg T Proserpio V Clare S Speak AO Dougan G. T cell fate and clonality inference from single cell transcriptomes. Nat Methods. (2016) 13:329–32. 10.1038/nmeth.3800 26950746 PMC4835021

[19] Lindeman I Emerton G Mamanova L Snir O Polanski K Qiao S-W. BraCeR: B-cell-receptor reconstruction and clonality inference from single-cell RNA-seq. Nat Methods. (2018) 15:563–5. 10.1038/s41592-018-0082-3 30065371

[20] Eltahla AA Rizzetto S Pirozyan MR Betz-Stablein BD Venturi V Kedzierska K. Linking the T cell receptor to the single cell transcriptome in antigen-specific human T cells. Immunol Cell Biol. (2016) 94:604–11. 10.1038/icb.2016.16 26860370

[21] Rizzetto S Koppstein DNP Samir J Singh M Reed JH Cai CH. B-cell receptor reconstruction from single-cell RNA-seq with VDJPuzzle. Bioinformatics. (2018) 34:2846–7. 10.1093/bioinformatics/bty203 29659703

[22] Canzar S Neu KE Tang Q Wilson PC Khan AA. BASIC: BCR assembly from single cells. Bioinformatics. (2017) 33:425–7. 10.1093/bioinformatics/btw631 28172415 PMC5408917

[23] Azizi E Carr AJ Plitas G Cornish AE Konopacki C Prabhakaran S. Single-cell map of diverse immune phenotypes in the breast tumor microenvironment. Cell. (2018) 174:1293–308.e36. 10.1016/j.cell.2018.05.060 29961579 PMC6348010

[24] Arguel MJ LeBrigand K Paquet A Ruiz García S Zaragosi LE Barbry P A cost effective 5′ selective single cell transcriptome profiling approach with improved UMI design. Nucleic Acids Res. (2017) 45:e48 10.1093/nar/gkw1242 27940562 PMC5397152

[25] Tang DTP Plessy C Salimullah M Suzuki AM Calligaris R Gustincich S. Suppression of artifacts and barcode bias in high-throughput transcriptome analyses utilizing template switching. Nucleic Acids Res. (2013) 41:e44. 10.1093/nar/gks1128 23180801 PMC3562004

[26] Macosko EZ Basu A Satija R Nemesh J Shekhar K Goldman M. Highly parallel genome-wide expression profiling of individual cells using nanoliter droplets. Cell. (2015) 161:1202–14. 10.1016/j.cell.2015.05.002 26000488 PMC4481139

[27] Victora GD Dominguez-Sola D Holmes AB Deroubaix S Dalla-Favera R Nussenzweig MC. Identification of human germinal center light and dark zone cells and their relationship to human B-cell lymphomas. Blood. (2012) 120:2240–8. 10.1182/blood-2012-03-415380 22740445 PMC3447782

[28] Seifert M Przekopowitz M Taudien S Lollies A Ronge V Drees B. Functional capacities of human IgM memory B cells in early inflammatory responses and secondary germinal center reactions. Proc Natl Acad Sci USA. (2015) 112:E546–55. 10.1073/pnas.1416276112 25624468 PMC4330750

[29] Bacher P Schink C Teutschbein J Kniemeyer O Assenmacher M Brakhage AA. Antigen-reactive T cell enrichment for direct, high-resolution analysis of the human naive and memory Th cell repertoire. J Immunol. (2013) 190:3967–76. 10.4049/jimmunol.1202221 23479226

[30] Bacher P Hohnstein T Beerbaum E Röcker M Blango MG Kaufmann S. Human anti-fungal Th17 immunity and pathology rely on cross-reactivity against *Candida albicans*. Cell. (2019) 176:1340–55.e15. 10.1016/j.cell.2019.01.041 30799037

[31] Picelli S Björklund ÅK Faridani OR Sagasser S Winberg G Sandberg R. Smart-seq2 for sensitive full-length transcriptome profiling in single cells. Nat Methods. (2013) 10:1096–8. 10.1038/nmeth.2639 24056875

[32] Ding J Adiconis X Simmons SK Kowalczyk MS Hession CC Marjanovic ND Systematic comparative analysis of single cell RNA-sequencing methods. bioRxiv. (2019) 2019:632216 10.1101/632216

[33] Li H Leun AM van der Y I Lubling Y Gelbard-Solodkin D Akkooi ACJ. Dysfunctional CD8 T cells form a proliferative, dynamicallyregulated compartment within human melanoma. Cell. (2018) 176:775–89.e18. 10.1016/j.cell.2018.11.043 30595452 PMC7253294

[34] von Boehmer L Liu C Ackerman S Gitlin AD Wang Q Gazumyan A. Sequencing and cloning of antigen-specific antibodies from mouse memory B cells. Nat Protoc. (2016) 11:1908–23. 10.1038/nprot.2016.102 27658009

[35] Zajac P Islam S Hochgerner H Lönnerberg P Linnarsson S. Base preferences in non-templated nucleotide incorporation by MMLV-derived reverse transcriptases. PLOS ONE. (2013) 8:e85270. 10.1371/journal.pone.0085270 24392002 PMC3877366

[36] Islam S Zeisel A Joost S La Manno G Zajac P Kasper M. Quantitative single-cell RNA-seq with unique molecular identifiers. Nat Methods. (2014) 11:163–6. 10.1038/nmeth.2772 24363023

[37] Jaitin DA Kenigsberg E Keren-Shaul H Elefant N Paul F Zaretsky I. Massively parallel single-cell RNA-seq for marker-free decomposition of tissues into cell types. Science. (2014) 343:776–9. 10.1126/science.1247651 24531970 PMC4412462

[38] Keren-Shaul H Kenigsberg E Jaitin DA David E Paul F Tanay A. MARS-seq2.0: an experimental and analytical pipeline for indexed sorting combined with single-cell RNA sequencing. Nat Protoc. (2019) 14:1841–62. 10.1038/s41596-019-0164-4 31101904

[39] Yost KE Satpathy AT Wells DK Qi Y Wang C Kageyama R. Clonal replacement of tumor-specific T cells following PD-1 blockade. Nat Med. (2019) 25:1251–9. 10.1038/s41591-019-0522-3 31359002 PMC6689255

[40] Mimitou EP Cheng A Montalbano A Hao S Stoeckius M Legut M Expanding the CITE-seq tool-kit: detection of proteins, transcriptomes, clonotypes and CRISPR perturbations with multiplexing, in a single assay. Nat Methods. (2019) 16:409–12. 10.1038/s41592-019-0392-0 31011186 PMC6557128

[41] Renand A Archila LD McGinty J Wambre E Robinson D Hales BJ. Chronic cat-allergen exposure induces a TH2 cell-dependent IgG4 response related to low-sensitization. J Allergy Clin Immunol. (2015) 136:1627–35.e13. 10.1016/j.jaci.2015.07.031 26371841 PMC4679456

[42] Stuart T Butler A Hoffman P Hafemeister C Papalexi E Mauck WM. Comprehensive integration of single-cell data. Cell. (2019) 177:1888–902.e21. 10.1016/j.cell.2019.05.031 31178118 PMC6687398

[43] Kowalczyk MS Tirosh I Heckl D Rao TN Dixit A Haas BJ. Single-cell RNA-seq reveals changes in cell cycle and differentiation programs upon aging of hematopoietic stem cells. Genome Res. (2015) 25:1860–72. 10.1101/gr.192237.115 26430063 PMC4665007

[44] Attaf-Bouabdallah N Cervera-Marzal I Dong C Gil L Renand A Spinelli L FB5P-seq: FACS-based 5-prime end single-cell RNAseq for integrative analysis of transcriptome and antigen receptor repertoire in B and T cells. bioRxiv [Preprint]. (2019) 795575 10.1101/795575

