# Corrigendum: FB5P-seq: FACS-Based 5-Prime End Single-Cell RNA-seq for Integrative Analysis of Transcriptome and Antigen Receptor Repertoire in B and T Cells

**DOI:** 10.3389/fimmu.2020.01521

**Published:** 2020-07-17

**Authors:** Noudjoud Attaf, Iñaki Cervera-Marzal, Chuang Dong, Laurine Gil, Amédée Renand, Lionel Spinelli, Pierre Milpied

**Affiliations:** ^1^Aix Marseille Université, CNRS, INSERM, Centre d'Immunologie de Marseille-Luminy, Marseille, France; ^2^Centre de Recherche en Transplantation et Immunologie UMR1064, INSERM Université de Nantes, Nantes, France

**Keywords:** single-cell RNA sequencing, transcriptome, antigen receptor, B cells, T cells

In the original article, there was a mistake in Table S3 as published. The sequence of Read2_SP, the Read2 sequencing primer, was incorrect. The corrected Table S3 appears below.

**Table S3:** Primers used in FB5P-seq.

**Primer name**	**Function in FB5P-seq**	**Sequence 5^**′**^-3^**′**^**
(dT)30_Smarter	Prime Reverse Transcription at 3′ ends of polyadenylated mRNA and introduce PCR handle	TGCGGTATCTAAAGCGGTGAGTTTTTTTTTTTTTTTTTTTTTTTTTT TTTTVN
TSO_BCx_UMI5_TATA	Perform template switching and introduce 8 nt. well-specific barcode & 5 nt. UMI	AGACGTGTGCTCTTCCGATCTXXXXXXXXNNNNNTATArGrGrG
Satija_PCR	Forward primer for LD-PCR amplification of cDNA libraries	AGACGTGTGCTCTTCCGATCT
SmarterR	Reverse primer for LD-PCR amplification of cDNA libraries	TGCGGTATCTAAAGCGGTGAG
i7_BC1	Forward primer for 5′-end enrichment of tagmented library and incorporation of plate-specific barcode	CAAGCAGAAGACGGCATACGAGATCCTGGTAGGTGACTGGAG TTCAGACGTGTGCTCTTCCGATCT
i7_BC2		CAAGCAGAAGACGGCATACGAGATTAAGCATGGTGACTGGAG TTCAGACGTGTGCTCTTCCGATCT
i7_BC3		CAAGCAGAAGACGGCATACGAGATAGATGTGCGTGACTGGAG TTCAGACGTGTGCTCTTCCGATCT
i7_BC4		CAAGCAGAAGACGGCATACGAGATGTCGAGCAGTGACTGGAG TTCAGACGTGTGCTCTTCCGATCT
i7_BC5		CAAGCAGAAGACGGCATACGAGATGAATTGCTGTGACTGGAG TTCAGACGTGTGCTCTTCCGATCT
i7_BC6		CAAGCAGAAGACGGCATACGAGATAAGCAACTGTGACTGGAG TTCAGACGTGTGCTCTTCCGATCT
i7_primer	Forward primer for amplification of sequencing library	CAAGCAGAAGACGGCATACGA
Read1_SP	Read1 sequencing primer	TCGTCGGCAGCGTCAGATGTGTATAAGAGACAG
i7_SP	Index Read i7 sequencing primer	AGATCGGAAGAGCACACGTCTGAACTCCAGTCAC
Read2_SP	Read2 sequencing primer	GTGACTGGAGTTCAGACGTGTGCTCTTCCGATCT

The authors apologize for this error and state that this does not change the scientific conclusions of the article in any way. The original article has been updated.

