# Corrigendum: FB5P-seq: FACS-Based 5-Prime End Single-Cell RNA-seq for Integrative Analysis of Transcriptome and Antigen Receptor Repertoire in B and T Cells

**DOI:** 10.3389/fimmu.2020.02047

**Published:** 2020-09-03

**Authors:** Noudjoud Attaf, Iñaki Cervera-Marzal, Chuang Dong, Laurine Gil, Amédée Renand, Lionel Spinelli, Pierre Milpied

**Affiliations:** ^1^Aix Marseille Université, CNRS, INSERM, Centre d'Immunologie de Marseille-Luminy, Marseille, France; ^2^Centre de Recherche en Transplantation et Immunologie UMR1064, INSERM Université de Nantes, Nantes, France

**Keywords:** single-cell RNA sequencing, transcriptome, antigen receptor, B cells, T cells

In the original article, there was a mistake in Table S4 as published. The sequences of some of the barcodes were incorrect and corresponded to a functional, but non optimized, version of the protocol. The corrected Table S4 appears below.

**TABLE S4:** List of well-specific barcodes used in TSO_BCx_UMI5_TATA primers.

**Barcode Index**	**Barcoded Well**	**Barcode 5′-3′**	**Barcode Index**	**Barcoded Well**	**Barcode 5′-3′**
1	A1	CGTCTAAT	49	E1	AACATTCT
2	A2	AGACTCGT	50	E2	CTACGCTG
3	A3	GCACGTCA	51	E3	GGGATTGT
4	A4	TCAACGAC	52	E4	TGATGTAG
5	A5	ATTTAGCG	53	E5	TTCGCTGT
6	A6	ATACAGAC	54	E6	GAAGACTT
7	A7	TGCGTAGG	55	E7	TCTGGGCA
8	A8	TGGAGCTC	56	E8	TCGCTACA
9	A9	TGAATACC	57	E9	GTGTTAGC
10	A10	TCTCACAC	58	E10	ATGCGACG
11	A11	TACTGGTA	59	E11	GAGGGTAG
12	A12	ACGATAGG	60	E12	CGGGTGAA
13	B1	GATGTCGA	61	F1	GCCATCTT
14	B2	TTACGGGT	62	F2	TGCGACAT
15	B3	GAATGAGT	63	F3	TCTATGGT
16	B4	CTTTGACA	64	F4	AGGACTTA
17	B5	AGAGATCT	65	F5	CCGCTCAG
18	B6	GAGTCCTG	66	F6	ACTAGCGA
19	B7	CACACTGA	67	F7	GTAACTCC
20	B8	GTTACAGG	68	F8	CGGAAGTG
21	B9	GGACCTTT	69	F9	CCGAGTAC
22	B10	TAGACTAT	70	F10	GATCTGAG
23	B11	ACTGTTTG	71	F11	ACCTGGAG
24	B12	AAGTGGCT	72	F12	CATGGGTT
25	C1	TGCTCTCA	73	G1	ATTCCTAG
26	C2	CGGCGTGG	74	G2	TCGAACCG
27	C3	GTGCATGA	75	G3	TCCACACT
28	C4	GTCATTAG	76	G4	AGGTAAAG
29	C5	AGCTCCTT	77	G5	TAGGCGCG
30	C6	TCACCCGA	78	G6	ACAGGCAT
31	C7	GTTGCCAC	79	G7	TTTGTGTC
32	C8	CTAATGCG	80	G8	TGAGCATA
33	C9	AACGAGGT	81	G9	TTAGACGC
34	C10	AGCCACCA	82	G10	CGCTTGCT
35	C11	TAGTGAAC	83	G11	ATTGGAGC
36	C12	CCAGTCCA	84	G12	CATAGTCG
37	D1	ACCTCAGC	85	H1	TCTTGCTG
38	D2	GGTGGACT	86	H2	GGGACAAC
39	D3	CCGGCGTC	87	H3	ATATTCCC
40	D4	TAACTCCG	88	H4	TGTTAAGC
41	D5	ACACCGTG	89	H5	TACGCCTC
42	D6	GTAGAACG	90	H6	CACTTATC
43	D7	GGATTGAC	91	H7	ACCGCTAA
44	D8	ACGTATCC	92	H8	TAAGGTCC
45	D9	TTCGGAAA	93	H9	GAAAGGTG
46	D10	AGTTGTGT	94	H10	ACGTTGTA
47	D11	AAGCACAT	95	H11	GTCTGCCG
48	D12	CTGTCATT	96	H12	GCATTTGG

The authors apologize for this error and state that this does not change the scientific conclusions of the article in any way. The original article has been updated.

