# Supplementary material for: FB5P-seq: FACS-Based 5-Prime End Single-Cell RNA-seq for Integrative Analysis of Transcriptome and Antigen Receptor Repertoire in B and T Cells
Source: Front Immunol. 2020 Mar 3;11:216. doi: 10.3389/fimmu.2020.00216 (PMC7062913; doi:10.3389/fimmu.2020.00216)
Supplement: Supplementary file 1 [file Data_Sheet_1.pdf]

Supplementary Figures

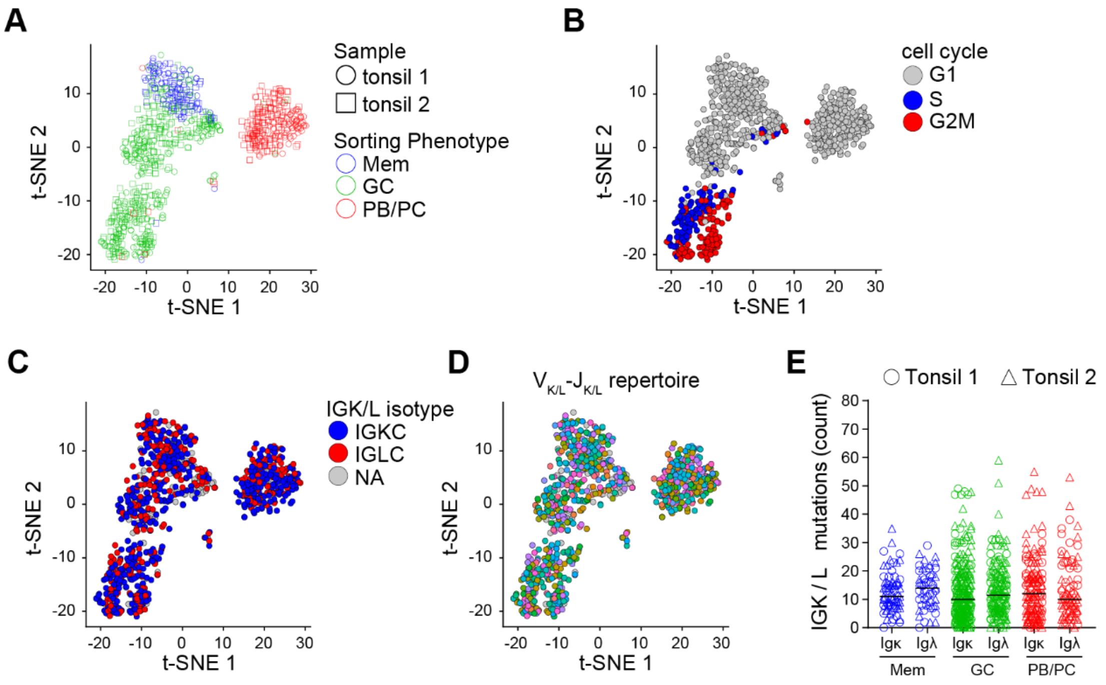

Figure S1. FB5P-seq analysis of human tonsil B cell subsets (related to Figure 4).

(A) t-SNE map of single human B cell subsets from Tonsil 1 (circles) and Tonsil 2 (squares) computed on 4,000 variable genes excluding BCR genes. Cells are colored based on sorting phenotype (n=845 cells). **(B-D)** t-SNE map of single human tonsil B cells colored by cell cycle phase **(B)**, BCR light chain isotype (NA: not applicable i.e. no IGK/L reconstructed) **(C),** or $V_{K/L}$-$J_{K/L}$ repertoire (grey cells: no IGK/L reconstructed) **(D)**. **(E)** Scatter plots showing IGK/L mutation frequency in human Tonsil 1 (circles) and Tonsil 2 (triangles) B cells sorted by their IGK/L isotype and phenotype (Mem B cells: n=71 Igκ+, n=51 Igλ+; GC B cells: n=253 Igκ+, n=163 Igλ+; PB/PCs: n=139 Igκ+, n=84 Igλ+).

Supplementary Tables

Sample	# plates	# cells sorted	# reads per cell (mean)	# cells after QC	% cells passing QC
Tonsil 1 B cells	5	420	540,087	386	91.9
Tonsil 2 B cells	6	504	525,890	459	91.1
CD4 T cells	1	84	397,670	82	97.6

Table S1. Quality control characteristics of FB5P-seq datasets.

Antibody target	Fluorochrome	Clone	Manufacturer	Final dilution
CD3	FITC	SK7	Biolegend	1/200
CD10	PE	HI10a	BD	1/20
CD14	FITC	HCD14	Biolegend	1/200
CD20	PE-Cy7	B9E9	Beckman Coulter	1/40
CD27	BV421	M-T271	BD	1/20
CD38	BV785	HIT2	BD	1/20
CD83	PE-Dazzle594	HB15e	Biolegend	1/50
CXCR4	APC	12G5	BD	1/5
IgD	FITC	Polyclonal Goat F(ab')2	Invitrogen	1/100

Table S2. List of antibodies for FACS analysis of tonsil B cells.

Primer name	Function in FB5P-seq	Sequence 5’-3’
(dT)30_Smarter	Prime Reverse Transcription at 3' ends of polyadenylated mRNA and introduce PCR handle	TGCGGTATCTAAAGCGGTGAGTTTTTTTTTTTTTTTTTTTTTTTTTTTTTVN
TSO_BCx_UMI5_TATA	Perform template switching and introduce 8 nt. well-specific barcode & 5 nt. UMI	AGACGTGTGCTCTTCCGATCTXXXXXXXXNNNNNTATArGrGrG
Satija_PCR	Forward primer for LD-PCR amplification of cDNA libraries	AGACGTGTGCTCTTCCGATCT
SmarterR	Reverse primer for LD-PCR amplification of cDNA libraries	TGCGGTATCTAAAGCGGTGAG
i7_BC1	Forward primer for 5’-end enrichment of tagmented library and incorporation of plate-specific barcode	CAAGCAGAAGACGGCATACGAGATCCTGGTAGGTGACTGGAGTTCAGACGTGTGCTCTTCCGATCT
i7_BC2		CAAGCAGAAGACGGCATACGAGATTAAGCATGGTGACTGGAGTTCAGACGTGTGCTCTTCCGATCT
i7_BC3		CAAGCAGAAGACGGCATACGAGATAGATGTGCGTGACTGGAGTTCAGACGTGTGCTCTTCCGATCT
i7_BC4		CAAGCAGAAGACGGCATACGAGATGTCGAGCAGTGACTGGAGTTCAGACGTGTGCTCTTCCGATCT
i7_BC5		CAAGCAGAAGACGGCATACGAGATGAATTGCTGTGACTGGAGTTCAGACGTGTGCTCTTCCGATCT
i7_BC6		CAAGCAGAAGACGGCATACGAGATAAGCAACTGTGACTGGAGTTCAGACGTGTGCTCTTCCGATCT
i7_primer	Forward primer for amplification of sequencing library	CAAGCAGAAGACGGCATACGA
Read1_SP	Read1 sequencing primer	TCGTCGGCAGCGTCAGATGTGTATAAGAGACAG
i7_SP	Index Read i7 sequencing primer	AGATCGGAAGAGCACACGTCTGAACTCCAGTCAC
Read2_SP	Read2 sequencing primer	GTGACTGGAGTTCAGACGTGTGCTCTTCCGATCT

Table S3. Primers used in FB5P-seq.

Barcode Index	Barcoded Well	Barcode 5'-3'	Barcode Index	Barcoded Well	Barcode 5'-3'
1	A1	CGTCTAAT	49	E1	AACATTCT
2	A2	AGACTCGT	50	E2	CTACGCTG
3	A3	GCACGTCA	51	E3	GGGATTGT
4	A4	TCAACGAC	52	E4	TGATGTAG
5	A5	ATTTAGCG	53	E5	TTCGCTGT
6	A6	ATACAGAC	54	E6	GAAGACTT
7	A7	TGCGTAGG	55	E7	TCTGGGCA
8	A8	TGGAGCTC	56	E8	TCGCTACA
9	A9	TGAATACC	57	E9	GTGTTAGC
10	A10	TCTCACAC	58	E10	ATGCGACG
11	A11	TACTGGTA	59	E11	GAGGGTAG
12	A12	ACGATAGG	60	E12	CGGGTGAA
13	B1	GATGTCGA	61	F1	GCCATCTT
14	B2	TTACGGGT	62	F2	TGCGACAT
15	B3	GAATGAGT	63	F3	TCTATGGT
16	B4	CTTTGACA	64	F4	AGGACTTA
17	B5	AGAGATCT	65	F5	CCGCTCAG
18	B6	GAGTCCTG	66	F6	ACTAGCGA
19	B7	CACACTGA	67	F7	GTAACTCC
20	B8	GTTACAGG	68	F8	CGGAAGTG
21	B9	GGACCTTT	69	F9	CCGAGTAC
22	B10	TAGACTAT	70	F10	GATCTGAG
23	B11	ACTGTTTG	71	F11	ACCTGGAG
24	B12	AAGTGGCT	72	F12	CATGGGTT
25	C1	TGCTCTCA	73	G1	ATTCCTAG
26	C2	CGGCGTGG	74	G2	TCGAACCG
27	C3	GTGCATGA	75	G3	TCCACACT
28	C4	GTCATTAG	76	G4	AGGTAAAG
29	C5	AGCTCCTT	77	G5	TAGGCGCG
30	C6	TCACCCGA	78	G6	ACAGGCAT
31	C7	GTTGCCAC	79	G7	TTTGTGTC
32	C8	CTAATGCG	80	G8	TGAGCATA
33	C9	AACGAGGT	81	G9	TTAGACGC
34	C10	AGCCACCA	82	G10	CGCTTGCT
35	C11	TAGTGAAC	83	G11	ATTGGAGC
36	C12	CCAGTCCA	84	G12	CATAGTCG
37	D1	ACCTCAGC	85	H1	TCTTGCTG
38	D2	GGTGGACT	86	H2	GGGACAAC
39	D3	CCGGCGTC	87	H3	ATATTCCC
40	D4	TAACTCCG	88	H4	TGTTAAGC
41	D5	ACACCGTG	89	H5	TACGCCTC
42	D6	GTAGAACG	90	H6	CACTTATC
43	D7	GGATTGAC	91	H7	ACCGCTAA
44	D8	ACGTATCC	92	H8	TAAGGTCC
45	D9	TTCGGAAA	93	H9	GAAAGGTG
46	D10	AGTTGTGT	94	H10	ACGTTGTA
47	D11	AAGCACAT	95	H11	GTCTGCCG
48	D12	CTGTCATT	96	H12	GCATTTGG

Table S4. List of well-specific barcodes used in TSO_BCx_UMI5_TATA primers.
